# Tryptophan Metabolites and Aryl Hydrocarbon Receptor in Severe Acute Respiratory Syndrome, Coronavirus-2 (SARS-CoV-2) Pathophysiology

**DOI:** 10.3390/ijms22041597

**Published:** 2021-02-05

**Authors:** George Anderson, Annalucia Carbone, Gianluigi Mazzoccoli

**Affiliations:** 1 CRC Scotland & London, Eccleston Square, London SW1V 1PX, UK; anderson.george@rocketmail.com; 2 Department of Medical Sciences, Division of Internal Medicine and Chronobiology Laboratory, Fondazione IRCCS “Casa Sollievo della Sofferenza”, 71013 San Giovanni Rotondo, Italy; annalucia.carbone@gmail.com

**Keywords:** tryptophan, aryl hydrocarbon receptor, severe acute respiratory syndrome, SARS-CoV-2, COVID-19

## Abstract

The metabolism of tryptophan is intimately associated with the differential regulation of diverse physiological processes, including in the regulation of responses to severe acute respiratory syndrome, coronavirus-2 (SARS-CoV-2) infection that underpins the COVID-19 pandemic. Two important products of tryptophan metabolism, viz kynurenine and interleukin (IL)4-inducible1 (IL41)-driven indole 3 pyruvate (I3P), activate the aryl hydrocarbon receptor (AhR), thereby altering the nature of immune responses to SARS-CoV-2 infection. AhR activation dysregulates the initial pro-inflammatory cytokines production driven by neutrophils, macrophages, and mast cells, whilst AhR activation suppresses the endogenous antiviral responses of natural killer cells and CD8+ T cells. Such immune responses become further dysregulated by the increased and prolonged pro-inflammatory cytokine suppression of pineal melatonin production coupled to increased gut dysbiosis and gut permeability. The suppression of pineal melatonin and gut microbiome-derived butyrate, coupled to an increase in circulating lipopolysaccharide (LPS) further dysregulates the immune response. The AhR mediates its effects via alterations in the regulation of mitochondrial function in immune cells. The increased risk of severe/fatal SARS-CoV-2 infection by high risk conditions, such as elderly age, obesity, and diabetes are mediated by these conditions having expression levels of melatonin, AhR, butyrate, and LPS that are closer to those driven by SARS-CoV-2 infection. This has a number of future research and treatment implications, including the utilization of melatonin and nutraceuticals that inhibit the AhR, including the polyphenols, epigallocatechin gallate (EGCG), and resveratrol.

## 1. Introduction

There is a growing appreciation that tryptophan and its metabolites are crucial aspects of the severe acute respiratory syndrome, coronavirus-2 (SARS-CoV-2) pathophysiology that has driven the COVID-19 pandemic. SARS-CoV-2 severity and fatality are strongly driven by advanced age as well as pre-existing co-morbidities, such as obesity and type 2 diabetes (T2D), as well as by stress-associated conditions, including racial discrimination stress [1]. It is widely accepted that it is alterations in the immune response that drive an individual’s susceptibility to severe SARS-CoV-2 infection. This is supported by the World Health Organization (WHO), which recently admitted that their recommended treatments, viz Remdesivir, Lopinavir, β-interferon (IFN), and Hydroxychloroquine proved of little utility, whilst treatments more directly targeting the immune response, such as Dexamethasone, were more beneficial [2].

Tryptophan and its metabolites, including kynurenine and indole-3-pyruvate (I3P), can be differentially regulated over the course of SARS-CoV-2 infection, which is strongly determined by the influence of pre-existing high-risk conditions, such as obesity/T2D and ageing, on the immune response [1]. The influence of the tryptophan metabolites, kynurenine and 13P, are mediated via the activation of the aryl hydrocarbon receptor (AhR). The AhR can have a number of complex effects, that may be partly ligand-dependent as well as from the complex interactions of the AhR with metabolic and circadian processes [3].

AhR activation can dysregulate the immune response, contributing to an increase in the initial pro-inflammatory cytokine wave/storm to SARS-CoV-2. It is the heightened and prolonged initial ‘cytokine storm’ that underpins SARS-CoV-2 driven severity and fatality [4]. Increased AhR activation contributes to this heightened and prolonged ‘cytokine storm’. In the normal course of a viral infection, such initial macrophage, neutrophil, and mast cell-driven inflammatory processes are eventually superseded by the endogenous anti-viral cells, especially natural killer (NK) cells and CD8+ T cells [1]. NK cells and CD8+ T cells target and kill viral infected cells by mechanisms similar to how these cells kill and remove cancer cells. AhR activation on NK cells and CD8+ T cells leads to a state of ‘exhaustion’, which prevents or suppresses their capacity to eliminate virus-infected cells or cancer cells [5,6]. Consequently, the differential regulation of tryptophan metabolites to increase AhR ligands, such as kynurenine and I3P, are important determinants of SARS-CoV-2 infection severity and fatality.

**Figure 1:**
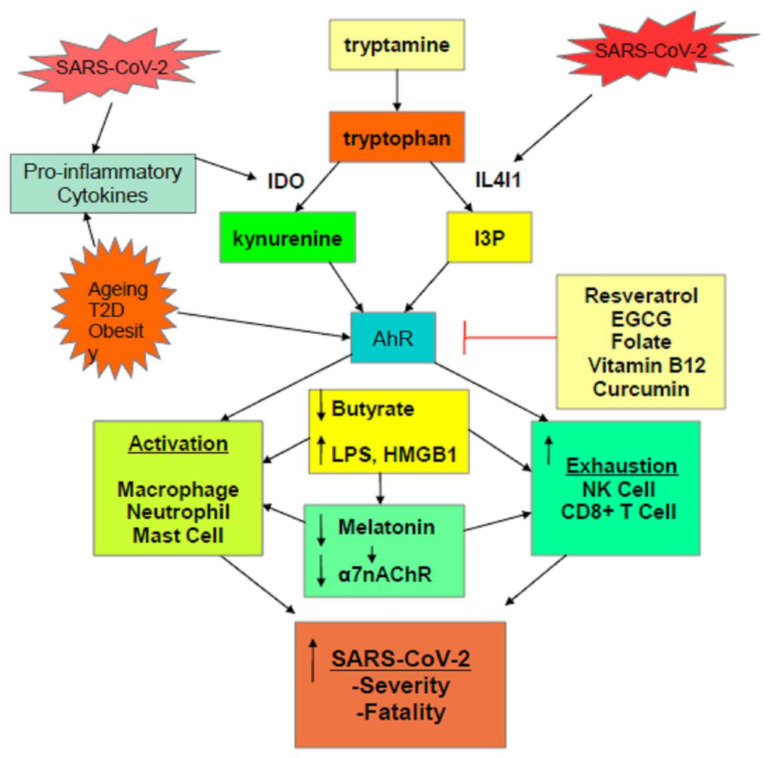
Scheme showing how the SARS-CoV-2 virus, and pre-existent high-risk medical conditions, shift tryptophan metabolism to increase AhR ligands. The activation of the AhR alters the nature of the initial ‘cytokine storm’ and suppresses the endogenous antiviral responses of NK cells and CD8+ T cells, leading to a prolonged activation of macrophages, neutrophils, and mast cells, as evident in severe SARS-CoV-2 infection. The driving to tryptophan to kynurenine and I3P, along with the elevated pro-inflammatory cytokines of the ‘cytokine storm’, also suppresses pineal melatonin production and therefore the induction of the α7nAChR by melatonin, thereby contributing lost vagal dampening of immune activity and raising sympathetic nervous system activation. The elevation in pro-inflammatory cytokines also increases gut dysbiosis/permeability, leading to a decrease in butyrate and raising LPS levels, further contributing to metabolic dysregulation of patterned immune responses to SARS-CoV-2 infection. A number of readily available nutraceuticals, such as resveratrol, EGCG, folate, vitamin B12, and curcumin, by AhR inhibition, may act to modulate how many processes influence SARS-CoV-2 pathophysiology.

The present article reviews data on the role of tryptophan and tryptophan metabolites in driving SARS-CoV-2 infection severity via AhR activation in immune cells. The driving of tryptophan to kynurenine and I3P decreases the availability of tryptophan for serotonin synthesis. As such, SARS-CoV-2 driven processes are linked to a decrease in serotonin and serotonin-derived N-acetylserotonin (NAS) and melatonin. The inhibition of the melatonergic pathway may therefore be an intimate aspects of SARS-CoV-2 pathophysiology, which is proposed to arise from alterations in immune cell metabolism [1] (Figure 1).

## 2. Tryptophan Metabolites and the Aryl Hydrocarbon Receptor

Although tryptophan is classically associated with being the necessary precursor for serotonin synthesis, the majority (95%) of the body’s tryptophan is converted to kynurenine by indoleamine 2,3-dioxygenase (IDO) and tryptophan 2,3-dioxygenase (TDO). An increase in pro-inflammatory cytokines, including IL-1β, IL-6, IL-18, and tumor necrosis factor (TNF), but especially IFNγ, increases IDO (and/or TDO in some cells) to further raise kynurenine levels and suppress serotonin, NAS, and melatonin levels [7]. TDO may also be increased by stress-associated cortisol and hypothalamus–pituitary–adrenal (HPA) axis activation [8]. As such, many medical conditions that are associated with an increase in pro-inflammatory cytokine or stress-induced dysregulation of the HPA axis will have increased IDO/kynurenine/AhR activation as an aspect of their pathophysiology, as evident in cancers [9], Alzheimer’s disease [10], depression [11], and arthritis [12], as well as in many other medical conditions. Consequently, the ‘cytokine storm’ that occurs to many viral infections, including influenza and SARS-CoV-2, will be associated with the induction of IDO/TDO/kynurenine/AhR activation and therefore wills alterations in the immune response and decreases in the serotonergic and melatonergic pathway. This is supported by data showing circulating kynurenine to be significantly increased in SARS-CoV-2 infection, in correlation with levels of severity [13].

However, there is another route whereby alterations in tryptophan metabolism may induce ligands that activate the AhR. Many bacterial and viral infections have been shown to increase interleukin-4-induced 1 (IL4I1), especially in macrophages [14,15], with IL4I1 leading to the induction of other AhR ligands, especially via I3P production. This would suggest that SARS-CoV-2 infection may be mediating some of its effects via the upregulation of IDO/kynurenine-independent AhR ligands. This is given some support by data showing that SARS-CoV-2 can increases AhR activation in an IDO-independent manner [16]. IL4I1 can be upregulated by a number of other immune cells, including dendritic cells, CD4+ T cells γδ (γδT-cells), and B-lymphocytes [17,18], although to a lesser extent than in macrophages. The IL4I1 induction in macrophages does not seem to have any suppressive impact on macrophage activation [17]. Rather, the effects of macrophage and dendritic cell IL4I1 and I3P induction are on the regulation of T and B lymphocytes [19], especially the suppression of CD3+, CD4+, and CD8+ T cells [18]. To date, there is no data on IL4I1 and I3P effects on NK cells, although it is clear that AhR activation is the major driver of ‘exhaustion’ and suppression of NK cells in the tumor microenvironment [3,6].

I3P and some of its derivates activate the AhR [20,21]. I3P can also generate indole-3-acetaldehyde (I3A), another AhR ligand, with both I3P and I3A being able to rearrange to the classical endogenous AhR ligand, 6-formylindolo(3,2-b)carbazole (FICZ), as well as the FICZ oxidation product, indolo(3,2-b)carbazole-6-carboxylic acid (CICZ) [22]. As such, the tryptophan metabolite pathway may be a rich source for AhR ligands that will be variably induced under different cellular conditions. It is also of note that the gut microbiome is an important source of I3P and I3A, with AhR activation in the gut being important to the maintenance of the gut barrier [23]. Future research will have to clarify as to whether I3P and/or its metabolites are AhR agonists.

## 3. Aryl Hydrocarbon Receptor

Classically, the humans AhR has been thought to function as a xenobiotic chemical sensor, and may still be referred to as the dioxin receptor. AhR activation aromatic (aryl) hydrocarbons is how the AhR derived its name. The AhR mediates many of its diverse and important effects via AhR activation induction of cytochrome P450 (CYP) metabolizing enzymes, including CYP1A, CYP1B1, and CYP1A2. CYP1A1 is important in the metabolism and regulation of estradiol, and therefore, in hormonal regulation, whilst CYP1B1 can O-demethylate melatonin to its immediate precursor, NAS, thereby driving alterations in the NAS/melatonin ratio, with some dramatic and contrasting consequences [24].

The AhR can be activated by a growing array of endogenous (e.g., FICZ), induced (e.g., kynurenine), and exogenous (e.g., air pollutants) ligands, highlighting its frequent involvement under a wide array of diverse circumstances. The AhR is highly expressed in the placenta and immune cells, with a wide array of diverse developmental effects. As indicated by AhR activation maintaining the gut barrier under challenge [23], the AhR has many beneficial effects, as well as detrimental effects when the production of AhR ligands becomes dysregulated. AhR activation leads to the induction of its own repressor, the AhR repressor (AHRR). The AhR is also differentially expressed over the circadian rhythm, indicating its involvement in wider systemic processes. It should also be noted that the AhR can be expressed in the mitochondrial membrane, although any direct effects on mitochondria regulation are still to be investigated [25].

The AhR is classified as one of a group of ‘basic helix-loop-helix’ transcription factors. Typically, the AhR is bound to, and inactivated by, a variety of chaperones in the cytoplasm. Upon ligand-binding, the AhR dissociates from these chaperones and nuclear translocates. In the nucleus, the AhR dimerizes with the AhR nuclear translocator (ARNT), where upon it then regulates numerous genes, including those expressing the xenobiotic-responsive element (XRE).

## 4. Gut Dysbiosis and Permeability in COVID-19

There is a growing appreciation of the role of the gut microbiome and gut permeability in the regulation of a host of diverse medical conditions. Such an array of consequences partly arises from two important process, viz: gut dysbiosis-associated decrease in the short-chain fatty acid, butyrate; and increased gut permeability-associated transfer of lipopolysaccharide (LPS) into the circulation. A decrease in butyrate and increase in circulating LPS has significant impacts on a wide array of cells, especially on the immune response.

Suppressed butyrate levels are evident over the course of SARS-CoV-2 infection [26], indicating a loss of butyrate’s histone deacetylase (HDAC) inhibitory activity as an aspect of SARS-CoV-2 infection. As butyrate and HDAC inhibition are important regulators of the immune response, with butyrate increasing the cytotoxicity and levels of NK cells in response to cancers [27], it would seem clear that such alterations in the gut microbiome short-chain fatty acid production are relevant to SARS-CoV-2 pathophysiology.

Hypertension is one of the high-risk conditions for severe/fatal SARS-CoV-2 infection, with a decrease in butyrate contributing to alterations in the gut–lung axis that contribute to hypertension-linked SARS-CoV-2 responses [28]. These authors indicate that butyrate’s inhibition of the high-mobility group box (HMGB)1 may be a crucial aspect of the protection afforded by butyrate, with decreased butyrate increasing the risk of SARS-CoV-2 symptom severity in people with hypertension [28]. However, as decreased butyrate production is an aspect of many medical conditions, including obesity and T2D [29], the optimization of butyrate production or supplementation with sodium butyrate is likely to afford protection in many of the high-risk medical conditions associated with increased SARS-CoV-2 severity/fatality [29]. Butyrate has also been found to increase a number of genes, which are regarded as anti-viral genes, thereby having wider anti-viral efficacy, including melatonin [30].

It should also be noted that gut-derived butyrate helps to prevent gut permeability, with effects that are partly mediated via an increase in mitochondrial function and associated induction of the melatonergic pathway [30]. Butyrate, like pineal melatonin, may ultimately act via the disinhibition of the pyruvate dehydrogenase complex (PDC), leading to an increased conversion of pyruvate to acetyl-CoA, thereby increasing ATP production from the tricarboxylic acid (TCA) cycle and oxidative phosphorylation (OXPHOS) as well as providing acetyl-CoA as a necessary co-factor for the first enzyme in the melatonergic pathway, arylalkylamine N-acetyltransferase (AANAT) [29]. As such, the effects of butyrate will be modulated by variations in the levels of tryptophan driven to serotonin production, given that serotonin is a necessary precursor for activation of the melatonergic pathway. Such effects of butyrate link gut dysbiosis and decreased butyrate production to gut permeability and increased circulating LPS levels.

By elevating circulating LPS, increased gut permeability modulates the patterning of the immune response via the activation of toll-like receptor (TLR)4 on different immune cells. Most viruses make their first host contact with mucosal surfaces, where bacterial microbials will already be well-established. Consequently, bacteria–virus interactions are an integral aspect of most viral infections [31]. However, an increase in circulating LPS, or other TLR4 agonists, such as gut-derived HMGB1 [32], can modulate the immune response to viral infection, including potentiating influenza virus lethality via effects in dendritic cells [33].

Clearly, alterations in the gut may act to regulate SARS-CoV-2 infection via variations in butyrate, LPS, and HMGB1. However, the gut and gut microbiome are also important to the uptake and metabolism of tryptophan, with the gut also being an important source for I3P, and therefore for AhR activation. In contrast to the many negative consequences of AhR activation in SARS-CoV-2 infection and in many other medical conditions, gut AhR activation helps to seal the gut barrier [23].

Overall, the gut is an important hub for many of the physiological factors and high-risk medical conditions associated with SARS-CoV-2 severity and fatality. Other factors known to regulate tryptophan metabolism, immunity, and SARS-CoV-2 infection may be acting, at least partly, via the gut. One such factor is vitamin D.

## 5. Vitamin D and COVID-19

A growing body of data shows vitamin D to afford protection against SARS-CoV-2 infection severity and fatality [34,35] ARS-CoV-2 infection outcome [36].

However, not everyone is convinced by the data indicating a role for vitamin D in SARS-CoV-2 infection severity/fatality [37], especially as lower vitamin D levels may interact with other non-measured and/or difficult to modify factors, such as age, obesity, ethnicity [38], and racial discrimination stress [1], in modulating immune responses to SARS-CoV-2 infection. It is long appreciated that vitamin D upregulates the levels and cytotoxicity of NK cells [39], with NK cells being important drivers of the endogenous anti-viral response to SARS-CoV-2 infection. Low vitamin D levels significantly correlate with reduced NK cell numbers and cytotoxicity in ICU and non-ICU SARS-CoV-2 infected patients with pneumonia [40]. Vitamin D also acts to suppress the heightened pro-inflammatory macrophage and myeloid derived suppressor cell contribution to the initial ‘cytokine storm’ during SARS-CoV-2 infection [41].

Vitamin D can also regulate mitochondrial function, including mitochondria ROS production and complexes II and IV, as shown in a variety of different cell types [42]. The effects of vitamin D on mitochondrial function, mitochondrial ROS, and levels of oxidative stress have recently been proposed to underpin the utility of vitamin D in the regulation of the SARS-CoV-2 infection [43]. It is also important to note the vitamin D receptor is also expressed in mitochondria [44], where it acts to regulate mitochondrial function and ROS production [45].

Importantly, vitamin D is a significant regulator of tryptophan metabolism. Murine data shows vitamin D to increase tryptophan hydroxylase (TPH)2, leading to a dramatic increase in neuronal serotonin production, whilst decreasing adipocyte leptin production [46]. Serotonin is a necessary precursor for the melatonergic pathway, and therefore in the regulation of metabolism and immune cell activation/deactivation [47], whilst heightened leptin levels correlate with SARS-CoV-2 infection severity on overweight patients [48]. As leptin influences the structural organization of NK cells [49], alterations in the regulation of leptin may be co-ordinated with wider regulation of the tryptophan/serotonergic/melatonergic pathway. Vitamin D also increases IDO in dendritic cells, thereby increasing the immune-suppressive levels of T-regulator (Treg) cells [50]. Alterations in Treg function may be an important aspect of immune dysregulation to severe SARS-CoV-2 infection [51].

As noted, vitamin D may also act via the gut, with vitamin D suppressing gut dysbiosis and increasing gut bacteria diversity [52]. Consequently, many of the effects of vitamin D on immune, mitochondria, epigenetic, and melatonergic pathway regulation may be mediated via effects in the gut. The prevention of gut permeability by vitamin D [53], may therefore be mediated via the upregulation of butyrate, whilst butyrate also acts to upregulate vitamin D receptor signaling [54]. Vitamin D may also suppress pro-inflammatory cytokine production [55], indicating that it may decrease pro-inflammatory cytokine-induced IDO, thereby impacting on the kynurenine/tryptophan ratio and inflammation driven AhR activation. However, as well as in dendritic cells, vitamin D can also upregulate the AhR in some cells [55]. This requires further investigation in different cell types, particularly as data in keratinocytes indicate that the hydroxyl-derivative of vitamin D3, the CYP11A1-derived 20,23(OH)_2_D3, is an AhR ligand [56]. The effects of vitamin D on AhR activation may be confounded by data showing that the vitamin D receptor can directly bind to an everted repeat (ER) 8 motif in the human CYP1A1 promoter [57]. CYP1A1 is typically used as an indicant of AhR activation, whilst it is CYP1B1 that seems responsible for the significant effects of AhR activation on mitochondrial and immune function [3]. HDAC inhibitors, such as butyrate, can suppress CYP11A1 [58], suggesting that variations in butyrate may act to regulate the induction of CYP11A1-derived 20,23(OH)_2_D3 and its activation of the AhR. The effects of butyrate, and other short-chain fatty acids, on CYP1B1 is context and cell dependent [59]. The interactions of the gut microbiome short-chain fatty acid, butyrate, acetate and propionate, via HDAC inhibition, on AhR-linked inductions will be important to determine in different cell types.

Such data indicate the complex interactions that vitamin D can have with other processes relevant to SARS-CoV-2 infection. For example, an increase in stress, including racial discrimination stress, may increase gut permeability and gut dysbiosis, with any decrease in butyrate attenuating butyrate’s potentiation of the vitamin D receptor. As such, as well as directly regulating gut permeability, vitamin D effects will be influenced by other factors, such as stressors, that increase gut dysbiosis/permeability.

The complexity of vitamin D receptor effects are further increased by data showing that melatonin binds to the vitamin D receptor and increases vitamin D-driven transcription [60]. This suggests that variations in pineal and local melatonin production may act to regulate vitamin D effects more directly, via melatonin interactions with the vitamin D receptor. It should be noted that melatonin may interact with, and regulate the activity of, a wide array of different receptors [61], considerably complicating its effects. Such data would indicate that the decrease in circadian, pineal melatonin with age may contribute to age-linked SARS-CoV-2 infection severity via alterations in melatonin’s regulation of the vitamin D receptor, and other receptors, as well as from the melatonin/Bmal1/SIRT1/SIRT3/PDC/acetyl-CoA/metabolism pathway. It will also be important to determine as to whether variations in mitochondrial and/or cytoplasmic melatonin production, as influenced by AhR/CYP1B1, act to modulate the mitochondrial vitamin D receptor. This could suggest differential effects of the mitochondrial vitamin D receptor under conditions of altered mitochondrial function that is co-ordinated with variations in the AhR regulation of the melatonergic pathway. As such, the variations in mitochondrial melatonergic pathway may then act to differential regulate the effects of vitamin D. Likewise variations in the circadian, pineal melatonin production may modulate the vitamin D receptor effects over the circadian rhythm.

## 6. Circadian Rhythm and COVID-19

The above factors, viz the AhR, vitamin D, the gut microbiome, and especially pineal melatonin, are all intimately linked to the circadian rhythm. Although the circadian rhythm and circadian genes have long been associated with the regulation of viral infections [62,63], there is a relative paucity of data on the role of the circadian rhythm in the SARS-CoV-2 infection. Sleep-wake disruption is a SARS-CoV-2 infection severity risk factor, including for people with dementia and diabetes [64,65]. It is of note that elderly age is the major risk factor for severity/fatality in the COVID-19 pandemic. People over 80 years of age show a 10-fold decrease in the levels of pineal melatonin production, suggesting that the loss of pineal melatonin over ageing may contribute to the association of SARS-CoV-2 infection severity with age. Ageing associated factors may contribute to the loss of pineal melatonin, including via increased levels of pro-inflammatory cytokines, amyloid-β and circulating LPS, all of which may act to inhibit pineal melatonin production. The loss of pineal melatonin is important, as melatonin at night acts to reset the cells of the immune system, thereby better optimizing immune cell function during daytime, when the immune system is more likely to be under challenge.

Immunosenescence is widely used to explain the catastrophic changes that can arise in dementia and the general inability of the elderly to resist immune challenge [66]. Recent work has indicated that the loss of pineal melatonin may underpin immunosenescence via the lost/suppressed night-time resetting of immune cell metabolism [29]. All immune cells require the upregulation of glycolysis, coupled to maintained OXPHOS, in order to become activated. By shifting cells from glycolytic metabolism to OXPHOS, pineal melatonin dampens any lingering pro-inflammatory activity in the immune system, whilst optimizing immune cells for daytime challenge. This is achieved by melatonin’s induction of the circadian gene, Bmal1, and SIRT1, leading to a melatonin/Bmal1/SIRT1/SIRT3/PDC/acetyl-CoA pathway, whereby acetyl-CoA not only increases ATP from the TCA cycle and OXPHOS, but also allows for the activation of the mitochondrial and cytoplasmic melatonergic pathway. The latter arises from acetyl-CoA being a necessary co-substrate for the initial enzyme in the melatonergic pathway, viz AANAT. Acetyl-CoA also dampens COX2-driven inflammatory activity by the acetylation and inhibition of COX2, as occurs with aspirin. Consequently, the loss of pineal melatonin will contribute to, if not drive, the changes arising in immunosenescence.

The efficacy of gut microbiome-derived butyrate is mediated via optimized mitochondrial function, arising from an increased PDC and associated melatonergic pathway activation [30]. As such, the effects of the gut microbiome-derived butyrate are intimately linked to the circadian alterations driven by pineal melatonin. Gut dysbiosis and associated increased gut permeability, by raising levels of the TLR4 ligands, LPS and HMGB1, will suppress pineal melatonin production [67]. Consequently, ageing-associated gut dysbiosis/permeability will contribute to the inhibition of pineal melatonin’s optimization and resetting of immune cell metabolism. As noted, the effects of vitamin D at the vitamin D receptor may be significantly determined by variations in pineal and local melatonin production [60]. The other major circadian factor highlighted above, the AhR, is in negative reciprocal interactions with melatonin [68].

Overall, such data highlights the important interactions of COVID-19 regulatory factors, such as the AhR, vitamin D, butyrate, amyloid-β, and LPS, with pineal melatonin and the circadian rhythm regulation of the immune system.

## 7. Integrating Tryptophan Metabolism into COVID-19 Pathophysiology

As indicated throughout, variations in the regulation of tryptophan and its metabolites are intimately linked to key processes underpinning SARS-CoV-2 pathophysiology. The raised levels of pro-inflammatory cytokines during the initial ‘cytokine’ storm will drive the upregulation of IDO and its conversion of tryptophan to kynurenine. This has two important consequences, viz increasing AhR activation and decreasing the availability of tryptophan for the serotonergic and melatonergic pathways. As SARS-CoV-2 may increase AhR ligands independently of IDO induction, it is proposed that SARS-CoV-2, like other viral infections, may be associated with the upregulation of IL4I1, and thereby with the driving of tryptophan to the production of other AhR ligands, viz I3P and I3A [16]. See Figure 1.

Increased AhR activation will dysregulate the immune response, contributing to a heightened pro-inflammatory phase during the initial ‘cytokine storm’, whilst also suppressing the endogenous antiviral cell responses of NK cells and CD8+ T cells. Consequently, there is prolongation of heightened pro-inflammatory activity in the initial phase of infection, at least in part driven by the suppressed antiviral responses of NK cells and CD8+ T cells, which would typically control the immune response around 7 days after initial infection. It has been recognized that this dysregulated immune response is the major driver of SARS-CoV-2 severity and fatality.

SARS-CoV-2 severity risk factors, including elderly age, obesity, and T2D, prime for an altered immune response by a number of mechanisms. These conditions are all associated with an increase in AhR levels and activity as well as an increase in pro-inflammatory cytokines/IDO/kynurenine, leading to AhR activation. Diet is known to regulate IL4I1 [69], suggesting that dietary factors contributing to obesity and T2D may be acting in part via the upregulation of IL4I1 and I3P.

As well as regulating the immune response, AhR activation primes platelets for activation, coagulation, and thrombin formation, as does TLR4 activation that may arise from gut permeability-derived LPS and HMGB1. Gut dysbiosis/permeability and AhR activation are therefore important contributors to the association of activated platelets to SARS-CoV-2 fatality. The gut is intimately associated with the regulation of tryptophan and its metabolites. Many of the beneficial effects of butyrate are mediated by its upregulation of the melatonergic pathway, highlighting that the driving of tryptophan to the production of AhR ligands and away from serotonin, NAS, and melatonin production, will attenuate the beneficial effects of butyrate. This may be of particular relevance in immune cells, where butyrate’s induction of PDC/acetyl-CoA/OXPHOS/TCA cycle/melatonergic pathway are important to a more optimized antiviral immune response by NK cells and CD8+ T cells.

The important role of the circadian rhythm may also be similarly modulated by variations in tryptophan regulation. Recent work would indicate that the importance of pineal melatonin to immune cell ‘resetting’ over the circadian rhythm may be mediated by the activation of the Bmal1/SIRT1/SIRT3/PDC/acetyl-CoA/OXPHOS/TCA cycle/melatonergic pathway [3]. The driving of tryptophan away from serotonin production will attenuate pineal melatonin’s influence on the metabolism of immune cells. Importantly, AhR activation arising from the induction of kynurenine and I3P will increase AhR-induced CYP1B1, thereby backward converting melatonin to NAS, whilst the ability of AhR activation to suppress 14-3-3 [70] can prevent the activation of the AANAT and the melatonergic pathway. 14-3-3 is necessary for the stabilization of AANAT, indicating that AhR suppression of 14-3-3 may inhibit mitochondrial and cytoplasmic melatonin production. As the release and autocrine effects of melatonin are required to switch immune cells from an M1-like pro-inflammatory phenotype to an M2-like, anti-inflammatory, pro-phagocytic phenotype [47], the induction of tryptophan metabolites can significantly alter the nature of the immune response. As such, as well as modulating circadian regulation of the immune system, an increase in tryptophan metabolite-driven AhR activation can change the nature of the activation–deactivation processes in individual immune cells.

There is a growing appreciation of the pathophysiological and treatment relevance of vitamin D in the SARS-CoV-2 infection. By regulating serotonin levels [46], vitamin D will modulate the serotonin-melatonergic pathway, whilst the suppression of pro-inflammatory cytokine production by vitamin D [55] will attenuate cytokine-induced IDO and the pro-inflammatory route for activation of the AhR by kynurenine. As noted, melatonin can bind the vitamin D receptor, thereby potentiating some vitamin D-driven transcription [60]. The suggests that attenuation of the tryptophan/serotonin/melatonin pathway by IDO, TDO, and IL4I1 can suppress vitamin D-driven transcription. As such, the effects of vitamin D in the SARS-CoV-2 infection may be intimately linked to alterations in tryptophan metabolism.

## 8. Future Research

Does SARS-CoV-2 increase IL4I1, and therefore the production of the AhR ligands, I3P and I3A?

Preclinical data show that prenatal/early postnatal exposure to AhR ligands leads to a reprogramming of the CD4+ and CD8+ T cell responses to viral infection, via the long-term maintenance of alterations in DNA methylation [71,72]. This is also supported by human data [73]. This could suggest that the upregulation of tryptophan-derived ligands, including kynurenine and I3P, in early development will modulate cytolytic cell responses to later infection. The relevance of AhR regulation of tryptophan metabolites in such processes, including via CYP1B1 regulation of the melatonergic pathway, will be important to determine. As many high-risk medical conditions for severe/fatal SARS-CoV-2 infection, including obesity and T2D [74], can have an early developmental etiology, the developmental overlaps of these conditions with alterations in responses to viral infection in later life will be important to determine.

Recent data show that even small elevations in plasma glucose associate with SARS-CoV-2 infection severity/fatality [75], indicating a role regulation of the gut microbiome within the context of the gut–liver and gut–brain axes [76], in modulating SARS-CoV-2 severity. The roles of the tryptophan metabolites, kynurenine and I3P, in the activation of the AhR in driving such glucose dysregulation [77], in concert with AhR-mediated dysregulation of the immune response, will be important to determine.

As to how the tryptophan metabolites interact with the alpha 7 nicotinic acetylcholine receptor (α7nAChR) in SARS-CoV-2 pathophysiology will be important to determine. The conversion of kynurenine to kynurenic acid (KYNA) may contribute to KYNA antagonism of the α7nAChR, as well as N-methyl-d-aspartate (NMDA) receptor antagonism. The α7nAChR can significantly inhibit pulmonary viral infections via effects in pulmonary epithelial cells as well as in the regulation of the immune response, reviewed in [29]. Importantly, the α7nAChR is regulated by melatonin [78], suggesting that the suppression of pineal, and perhaps local, melatonin in SARS-CoV-2 infection may contribute to a decrease in α7nAChR activation in pulmonary epithelial cells, and the immune system α7nAChR level and activity in SARS-CoV-2 infection will contribute to inflammation during the initial ‘cytokine storm’ [29,79]. The regulation of tryptophan metabolism may then directly modulate the α7nAChR via KYNA, which also activates the AhR, as well as via melatonin.

The α7nAChR is an important mediator of vagal nerve activity, and therefore in the sympathetic/parasympathetic balance of the autonomic nervous system. Many of the high-risk conditions associated with SARS-CoV-2 infection severity/fatality have heightened levels of sympathetic nervous system activity [80,81], which may be contributed by increased KYNA and decreased melatonin that suppresses α7nAChR activity and levels, respectively. The effects of KYNA and decreased melatonin on the α7nAChR may therefore be an aspect of high-risk medical conditions per se, as well as being driven by SARS-CoV-2 infection via tryptophan metabolite regulation. Clearly, disentangling the effects of α7nAChR activation from research focused on promoting anti-cigarette smoking [82] will be important for future research. It is also important that this is looked at in human cells, given the unique human duplicant dupα7 (CHRFAM7A), which negatively regulates the α7nAChR, and which can be independently regulated by different factors, including LPS, reviewed in [29]. As such, measurement of the role duplicant dupα7 (CHRFAM7A) in different experimental protocols will be necessary in order to clarify α7nAChR effects, if any.

The presence of pulmonary embolism in COVID-19 patients is high, with one Spanish study of consecutive hospitalized patients showing 35.6% of patients having a pulmonary embolism [83]. The role of I3P and kynurenine, via AhR activation, in priming platelets for elevated activity, coagulation, and thrombin production in severe SARS-CoV-2 infection [84], will be important to determine.

Given the paucity of SARS-CoV-2 data pertaining to many of the processes highlighted above, it is clear that many future research directions may be indicated. For example, the well-proven role of vitamin D in the regulation of immune responses may interact with the regulation of serotonin and melatonin production, as indicated above. The interactions of vitamin D with experiences of racial discrimination in modulating SARS-CoV-2 pathophysiology will be important to determine, including in tropical countries such as Brazil, where heightened levels of racial discrimination stress are evident. As such, vitamin D levels in equatorial countries may be in interaction with other important processes that occlude a simple interpretation of how vitamin D variations may interact with SARS-CoV-2 susceptibility and severity. As stressors, including possibly racial discrimination stress, can increase gut permeability and dysregulate the gut microbiome, whilst vitamin D also regulates gut permeability, it is clear that indicants of how vitamin D may modulate SARS-CoV-2 infection susceptibility and severity will need the recording of wider body systems. The acquisition of such data should provide a more detailed basis as to how tryptophan metabolites and the AhR interact with wider body systems.

## 9. Treatment Implications

Targeting aspects of tryptophan metabolism may have important prophylactic and treatment implications for the management of SARS-CoV-2 infection, particularly as the emergence of new variants would suggest that this virus is likely to have impacts for years, if not decades. Clearly, the suppression of infectivity and symptomatology by nutraceuticals is preferable to treatment with pharmaceuticals, given the lack of any efficacy of the four WHO recommended pharmaceuticals at the beginning of the COVID-19 pandemic [2].

## 10. Prophylactic Treatment

A number of studies have shown that people taking melatonin have up to a 64% decrease in the likelihood of getting SARS-CoV-2 infection, or have had no conscious symptoms [85]. The optimization of night-time melatonin production by 2–10 mg melatonin, about 20 min before bed-time is a readily achievable intervention. As melatonin is a natural product with no relevant side-effects and is widely used by millions of people worldwide, the utilization of melatonin as a prophylactic would not require extensive safety trials. The dramatic loss of pineal melatonin in the elderly and the proven utility of melatonin in the management of dementia, obesity, T2D would indicate that melatonin may have wider clinical benefits for people with high-risk conditions for SARS-CoV-2 severity/fatality.

Likewise, utilizing vitamin D supplements will have immune and gut microbiome/barrier integrity benefits as well as suppressing the pro-inflammatory cytokine response that drives tryptophan to the production of AhR ligands.

A number of nutraceuticals afford antagonism at the AhR, including green tea and its polyphenol, epigallocatechin gallate (EGCG). EGCG has also been shown to decrease SARS-CoV-2 entry into cells in vitro, with over two-times the efficacy of WHO recommended, Lopinavir [86,87].

Other AhR antagonists include resveratrol, vitamin B12, folate, and curcumin, all of which have wider health benefits, including via the regulation of immune function [16].

As melatonin may mediate some of its effects via increased α7nAChR, with the α7nAChR activation having possible prophylactic benefits in pulmonary epithelial cells, as in other viral infections [84], nicotine and/or pharmaceutical α7nAChR agonists, may have prophylactic utility. Data shows nicotine to have significant impacts on pulmonary epithelial cells, which could indicate some relevant impacts on SARS-CoV-2 infection [82], although it is highly likely that in vivo the effects of nAChR agonism will be in both immune cells and pulmonary epithelial cell interactions [88].

Pre- and pro-biotics, as well as dietary alterations, can slowly improve the α-diversity of gut microbiome, thereby increasing butyrate production. However, the utilization of the nutraceutical, sodium butyrate, will achieve optimal butyrate levels more quickly, whilst also increasing the production of butyrate-producing gut bacteria.

As with melatonin, such nutraceutical supplements will have wider benefits on the pathophysiology of SARS-CoV-2 high risk medical conditions, such as obesity and T2D.

## 11. Treating SARS-CoV-2 Infection

There is a growing appreciation of the potential clinical utility of melatonin in the treatment of SARS-CoV-2 infection. This has been indicated by data showing that patients needing intubation for SARS-CoV-2 infection are more likely to survive if they have been taking melatonin [89]. Such data highlight how the prophylactic use of melatonin, if unsuccessful in preventing infection, may still prove beneficial by decreasing the severity of any subsequent SARS-CoV-2 infection.

The results of an ongoing pilot study, looking at the utility of intravenous melatonin at 5–8 mg/kg body weight, in the treatment of severe SARS-CoV-2 infection [90], should clarify as to whether melatonin has treatment utility in the management of SARS-CoV-2 infection. Given the well-proven anti-inflammatory effects of melatonin, and its utilization by the body over the course of evolution to reset immune cells over the circadian rhythm as well as switching immune cells from and M1-like to an M2-like anti-inflammatory phenotype via its autocrine effects [47], it is likely that such high doses of melatonin will modulate the course of severe SARS-CoV-2 infection. Unlike dexamethasone, melatonin does not totally suppress the antiviral NK cells and CD8+ T cells, but rather seems to increase their cytotoxicity and antiviral efficacy, it may be that melatonin will have more utility than the limited efficacy of dexamethasone. Future research should clarify this in the near future, as well as to whether melatonin would have any impact on mitochondrial AhR regulation of mitochondrial transcription [25,91].

Melatonin also has negative reciprocal interactions with the AhR, suggesting that it may modulate some of the consequences of tryptophan metabolism dysregulation. However, it may be that more direct inhibition of the AhR over the course of SARS-CoV-2 infection will prove useful. For example, the dampening effects of melatonin on the initial ‘cytokine storm’ may be paired with AhR antagonism, e.g., via EGCG or resveratrol supplementation, in order to prevent the AhR antagonism/exhaustion in NK cells and CD8+ T cells. If of utility, the temporal utilization and dose of such AhR antagonists would have to be ascertained.

The utility of sodium butyrate, via its HDAC inhibitory capacity, induction of PDC and the melatonergic pathway [30], as well as its ability to increase the levels and cytotoxicity of NK cells requires investigation [27], including as to route and dose of administration.

## 12. Conclusions

Accumulating data on the pathophysiological changes driven by SARS-CoV-2 infection indicate an important role for variations in the metabolism of tryptophan. Pro-inflammatory cytokine induction of IDO, leads to kynurenine activation of the AhR, which can significantly suppress the endogenous antiviral responses of NK cells and CD8+ T cells, whilst dysregulating the initial pro-inflammatory cytokine responses of macrophages, neutrophils, and mast cells. Such processes are relevant in many other medical conditions, including as to how age associates with an increased susceptibility to a range of diverse medical conditions. As data show SARS-CoV-2 to have IDO/kynurenine-independent activation of the AhR, it is proposed that the SARS-CoV-2 virus, like some other viruses, activates IL4I1, thereby increasing the production of other AhR, such as I3P. As such, SARS-CoV-2 viral infection may have a number of routes to activate the AhR. The utilization of tryptophan to increase kynurenine in SARS-CoV-2 infection decreases the production of serotonin, and therefore suppresses the availability of serotonin as a necessary precursor for the melatonergic pathway. The cytokine suppression of pineal melatonin and the AhR-induced CYP1B1 suppression of local melatonin, leads to suboptimal melatonin regulation of mitochondrial metabolism, which underpins the dysregulated immune response to SARS-CoV-2 infection. This is further confounded by pro-inflammatory cytokine induced gut dysbiosis/permeability that suppresses butyrate and increases circulating LPS. Such a SARS-CoV-2 dysregulated immune responses, aided by pre-existent changes in high risk medical conditions, provide readily achievable treatment targets, including melatonin and AhR antagonists, such as EGCG, resveratrol, and curcumin, both as prophylactic and in the course of treatment of severe SARS-CoV-2 infection.

## Abbreviations

α7nAChR	alpha 7 nicotinic acetylcholine receptor
AANAT	aralkylamine N-acetyltransferase
AhR	aryl hydrocarbon receptor
ASMT	acetylserotonin methytransferase
CYP	cytochrome P450
dupα7	duplicating alpha 7nicotinic receptor
EGCG	epigallocatechin gallate
FICZ	6-formylindolo(3,2-b)carbazole
HDAC	histone deacetylase
HMGB	high-mobility group box
HPA	hypothalamic-pituitary-adrenal
I3A	indole-3-acetaldehyde
I3P	indole-3-pyruvate
IDO	indoleamine 2,3-dioxygenase
IFN	interferon
IL	interleukin
IL4I1	interleukin 4-inducible 1
KYNA	kynurenic acid
LPS	lipopolysaccharide
nAChR	nicotinic acetylcholine receptor
NAS	N-acetylserotonin
NK	natural killer cell
OXPHOS	oxidative phosphorylation
PDC	pyruvate dehydrogenase complex
SARS-CoV-2	severe acute respiratory disease, coronavirus 2
T2D	type II diabetes
TCA	tricarboxylic acid
TDO	tryptophan 2,3-dioxygenase
Th	T-helper
TLR	toll-like receptor
TNF	tumor necrosis factor
